# UK prescribing practice of anticoagulants in patients with chronic kidney disease: a nephrology and haematology-based survey

**DOI:** 10.1186/s12882-022-03041-w

**Published:** 2023-01-12

**Authors:** Kathrine Parker, Satarupa Choudhuri, Penny Lewis, Jecko Thachil, Sandip Mitra

**Affiliations:** 1 Manchester Institute of Nephrology and Transplantation, Manchester University NHS Foundation Trust, Oxford Road, M13 9WL Manchester, UK; 2 Division of pharmacy and optometry, School of health sciences, Manchester Academic Health Science Centre, The University of Manchester, University of Manchester, M13 9PT Manchester, UK; 3 Department of Haematology, Northern Care Alliance NHS Foundation Trust, Royal Oldham hospital, Rochdale Rd, OL1 2JH Oldham, UK; 4 Department of Haematology, Manchester University NHS Foundation Trust, Oxford Road, M13 9WL Manchester, UK; 5 Division of cardiovascular sciences, School of medical sciences, The University of Manchester, M13 9NT Manchester, UK

**Keywords:** Anticoagulation, Atrial fibrillation, Venous thromboembolism, Chronic kidney disease, Nephrotic syndrome

## Abstract

A survey to gain insight into anticoagulant prescribing practice in the setting of chronic kidney disease (CKD) across the UK was disseminated via renal and haematology networks. Areas of anticoagulant use included patients with venous thromboembolism (VTE), requiring thromboprophylaxis for VTE, Atrial Fibrillation (AF) and nephrotic syndrome.

An online-survey was disseminated via British Haematology Society, UK Kidney Association, and Renal Pharmacy Group over a five month period. All responses were voluntary and anonymous.

Among 117 responses there were 49 nephrology doctors, 47 renal pharmacists and 20 haematology clinicians. A specialist multidisciplinary team to discuss the specific anticoagulant management of these patients was only available to 3% (4/117) respondents. Renal function estimate used for anticoagulant dosing was mainly Cockcroft-Gault for pharmacists and haematology but lab-based estimates were used by nephrology doctors. Therapeutic dose of Low Molecular Weight Heparin was mostly likely to be reduced by one-third when used for VTE treatment, with the majority of units undertaking anti-Xa monitoring in CKD stage 5 and dialysis. Direct-acting Oral Anticoagulants are being used in patients with nephrotic syndrome, those with CKD stage 5 and on dialysis for VTE and AF in the absence of license in these indications.

This survey highlighted the significant differences between anticoagulant prescribing in CKD between two professional specialties and marked variation between centres in anticoagulant management strategies employed for these patients. With gaps still existing in the evidence base and answers to these not expected within the next few years, development of a best-practice guideline would be warranted to support clinicians in this field.

**Supplementary Information:**

The online version contains supplementary material available at 10.1186/s12882-022-03041-w.

## Background

Chronic Kidney Disease (CKD) is an increasing problem with an estimated 6.1% of the UK population over 16 years of age with CKD stages 3–5 (eGFR < 60ml/min/1.73m^2^) [1].

Patients with advanced kidney disease (CKD stages 3–5) have an increased risk of thrombotic events including deep vein thrombosis (DVT) and pulmonary embolism (PE) [2, 3]. This risk is related to a CKD-related platelet abnormalities, increases in procoagulants such as fibrinogen and Von Willebrand factor, endothelial dysfunction as well as other risk factors such as immobility, obesity and advancing age [4, 5]. Atrial Fibrillation (AF) and CKD often co-exist in patients with CKD with the incidence of AF increasing with decline in renal function [6, 7]. Thromboembolic events with AF can be reduced with the prescription of oral anticoagulants, which includes the vitamin K antagonists (VKAs) and direct oral acting anticoagulants (DOACs) [8]. However, the benefits of these agents are uncertain in advanced CKD due to an associated increased risk of bleeding [9], related to uraemia-induced platelet dysfunction and an increase in gastric angiodysplasias [10, 11].

Nephrotic syndrome (NS) is associated with high rates of thromboembolism, with the highest risk reported with membranous nephropathy [12]. The risks of thromboembolism are believed to be related to an imbalance in prothrombotic and antithrombotic factors and a reduction in thrombolytic activity [13]. However, studies of anticoagulant use in NS are limited [14, 15].

Historically, VKAs have been the mainstay of therapy for venous thromboembolism (VTE) and thromboembolic prevention in AF, with some use in NS. However, there are concerns with using VKAs in these populations in the CKD setting as maintaining INR in therapeutic range can be difficult, requires regular monitoring and can be associated with life-threatening calciphylaxis. Unfractionated heparin (UFH) has historically been used for the initial treatment of DVT and PE. Its complex monitoring and dosing regimens have meant that low molecular weight heparins (LMWH) are more widely used, however, exclusion of LMWHs from clinical trials in patients with a CrCl < 30ml/min has left uncertainty around dosing regime and monitoring requirements in these patients. With a standard dosing regime and reduced monitoring requirements, DOACs are currently the preferred oral anticoagulants in VTE and AF although patients with CrCl < 30ml/min were excluded from the DOAC randomised controlled studies (RCTs) [16–24]. Limited evidence informs anticoagulant practice in this population despite three of the DOACs being licensed to a CrCl 15ml/min in Europe. This national prescribing practice survey was undertaken to provide an insight into current use of anticoagulants in patients with CKD across specialities within the UK.

## Methods

### Data collection

An online questionnaire was developed by the study team including a renal pharmacist, nephrologists and haematologists. The questionnaire was designed to cover the most commonly encountered anticoagulant scenarios in kidney patients, which included management of acute VTE, prevention of thromboembolic events for AF and VTE thromboprophylaxis in medical patients and those with NS.

The questionnaire contained 21 questions with single or multiple responses (supplementary appendix 1). The questions referred to CKD the stages as defined by KDIGO. The responses were voluntary and anonymous.

The survey questions were inputted to Qualtrics™, which is an online survey tool allowing development, distribution and analysis of surveys. The survey was piloted among haematologists, nephrologists, pharmacists and a renal nurse prior to dissemination for clinical relevance and question interpretation.

### Participants

An e-mail invitation along with a link to the online survey were distributed from November 2021 to April 2022. Dissemination was via UK Kidney Association (UKKA) members including renal speciality registrars, Association of Nephrology Nurses UK (ANNUK) members, British Society for Haematology (BSH) members via the website, Haemstar networks of haematology registrars and via the haematology and renal Clinical Research Network (CRN) leads. The responses were anonymous and voluntary.

### Statistical analysis

Results were exported from Qualtrics™ as a Microsoft Excel file for analysis. The values are shown as numbers and percentages. In case of non-response for a particular question the number of responses is shown as n/N, where N is the total number of respondents. A two-sided *P*-value of < 0.05 was considered statistically significant. Due to the survey design, a descriptive statistical analysis was conducted.

## Results

A total of 117 responses were received from 39 different hospital trusts across the UK, supplementary Fig. 1 & supplementary Table 1. There were 46 respondents where response location could not be identified. Respondents included 49 nephrology doctors, 47 renal pharmacists (25% of all renal pharmacy group members), 20 haematology staff, Table 1. Four centres had responses from haematology, nephrology and renal pharmacy and eight centres had multiple responses from within the same professional group.

**Table 1:** Types of kidney patients seen by respondent speciality

	Haematology, n/N (%)	Nephrology doctors, n/N (%)	Renal Pharmacists, n/N (%)
VTE	18/20 (90)	45/49 (92)	47/47 (100)
VTE prophylaxis	17/20 (85)	48/49 (98)	47/47 (100)
AF	13/20 (65)	49/49 (100)	46/47 (98)
NS	9/20 (45)	48/49 (98)	46/47 (98)

### Multidisciplinary management

Only four respondents (3%) had access to multidisciplinary groups to discuss anticoagulant use in patients with CKD. These groups involved both nephrology and haematology input.

### Estimation of renal function for anticoagulant dosing

**Fig. 1:**
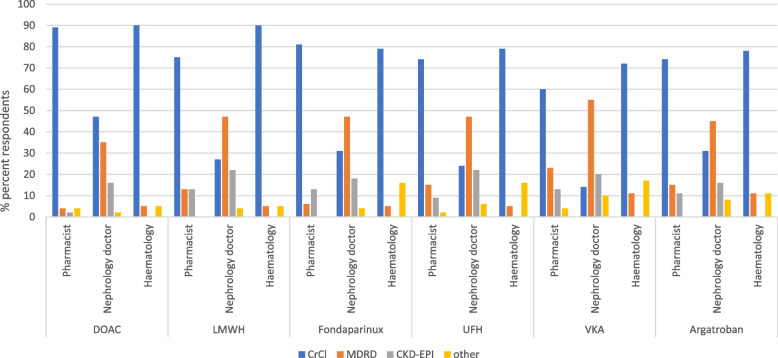
Renal function estimating equations used for anticoagulant dosing by different specialities. DOAC= Direct- acting oral anticoagulant, LMWH= Low molecular weight heparin, UFH= Unfractionated heparin, VKA= Vitamin K antagonist, CrCl= Cockroft-Gault creatinine clearance,  MDRD= Modified diet in renal disease, CKD-EPI = Chronic kidney disease epidemiology collaboration

There was variation in which renal function estimate used to guide anticoagulant dosing: nephrologists mainly used CKD-EPI, compared with pharmacists and haematologists who used Cockcroft-Gault Creatinine Clearance (C-G CrCl) for most anticoagulants, Fig. 1. For DOACs 89% of pharmacists and 90% of haematologists used C-G CrCl vs. 47% nephrology doctors, *p* < 0.0001. There was in-centre variation, in the use of renal function estimating equations used for anticoagulant dosing, between nephrologists in four centres.

### The use of anticoagulants by indication

####  Acute VTE treatment

For acute treatment of VTE the use of LMWH was the most frequent option to be selected by both nephrology and haematology professionals, Table 2. This was in preference to DOACs and UFH with the latter option being the least utilised.

**Table 2:** Anticoagulant treatment of acute VTE for patients with CKD by professional group

	Haematology, n/N (%)	Nephrology doctors, n/N (%)	Renal pharmacists, n/N (%)
DOACs	12/17 (71)	25/42 (60)	18/46 (39)
LMWH	14/17 (82)	40/42 (95)	44/46 (96)
UFH	4/17 (24)	14/42 (33)	0/46 (0)

####  Chronic VTE treatment

For the chronic treatment of VTE, among all professional groups DOACs were the most frequent therapeutic choice for patients with CKD stage 4. In patients with CKD stage 5 and on dialysis the use of VKAs were more likely, Table 3. In patients who were kidney transplant recipients (KTRs) nephrology professionals were more likely to prescribe DOACs with haematologists more likely to select warfarin.

**Table 3:** Venous thromboembolism treatment depending on CKD stage and professional group

	Haematology	Nephrology doctors	Renal pharmacists
CKD stage 4			
DOACs n/N (%)	9/13 (69%)	29/35 (83)	32/38 (84%)
LMWH n/N (%)	5/13 (38%)	7/35 (20)	19/38 (50%)
VKA n/N (%)	5/13 (38%)	19/35 (54)	27/38 (71%)
CKD stage 5			
DOACs n/N (%)	1/13 (8%)	15/35 (43%)	6/37 (16%)
LMWH n/N (%)	4/13 (31%)	8/35 (23%)	30/37 (81%)
VKA n/N (%)	10/13 (77%)	26/35 (74%)	30/37 (81%)
Dialysis			
DOACs n/N (%)	1/13 (8%)	14/35 (40%)	6/37 (16%)
LMWH n/N (%)	5/13 (38%)	9/35 (26%)	29/37 (78%)
VKA n/N (%)	9/13 (69%)	25/35 (71%)	32/37 (86%)
Kidney transplant			
DOACs n/N (%)	7/12 (58%)	29/35 (83%)	31/37 (84%)
LMWH n/N (%)	6/12 (50%)	8/35 (23%)	20/37 (54%)
VKA n/N (%)	9/12 (75%)	19/35 (54%)	26/37 (70%)

The preferred choice of DOAC was apixaban. All respondents who used DOACs for VTE treatment in CKD stage 5 and dialysis used apixaban. For patients with CKD stage 4 apixaban was used by 89% nephrology doctors, 67% of renal pharmacists and 57% of haematologists. There was some use of edoxaban and rivaroxaban. From the four centres where multiple professional groups responded, nephrology doctors used DOACs off-label for VTE in CKD stage 5 and dialysis in contrast to haematology and renal pharmacists. There was heterogeneity of dosing across respondents, supplementary Table 2.

### Choice of heparin for use in treatment of VTE

**Fig. 2:**
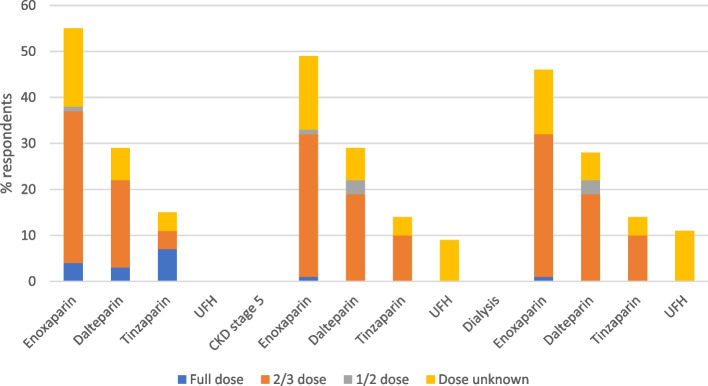
Heparin selection and dose used for various stages of CKD. From left to right CKD stage 4, CKD stage 5 and dialysis. UFH = Unfractionated heparin

For patients with CKD stage 4, 56% of respondents used enoxaparin, 29% used dalteparin and 16% used tinzaparin. This changed slightly for CKD 5 and dialysis as some units switched to using IV unfractionated heparin (UFH) with 9% and 11% of respondents using this respectively. The dosing strategies employed for each heparin across CKD stage are depicted in Fig. 2. For those respondents where LMWH dose reductions were stated the majority used a one third dose reduction in CKD stages 4 to dialysis.

### Monitoring of LMWH

**Fig. 3:**
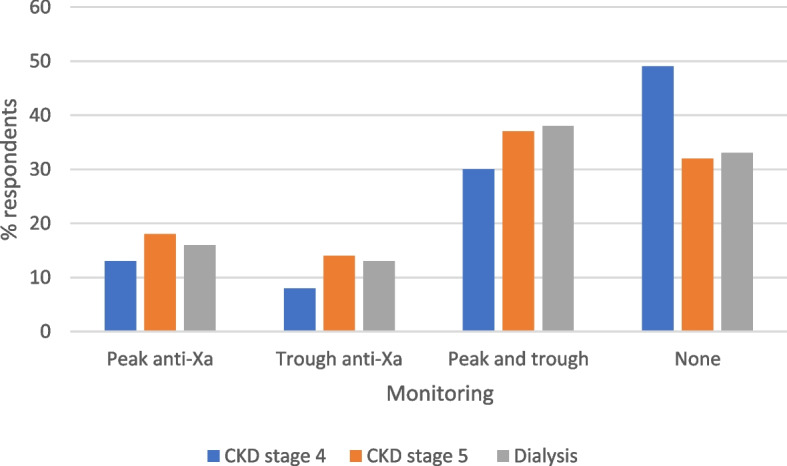
anti-Xa level monitoring undertaken in patients with CKD

There were 79 respondents to the question around anti-Xa level monitoring for CKD patients on LMWH. All haematologists responded that they would perform monitoring for patients with CKD on LMWH. There was variation across other professional groups as to what monitoring would be undertaken, shown in Fig. 3, with some respondents not undertaking any monitoring. Respondents were more likely to undertake monitoring for patients with CKD stages 5 and on dialysis with 67–68% undertaking monitoring compared to only 51% monitoring anti-Xa in patients with CKD stage 4. There was variation in one centre with two nephrology doctors undertaking anti-Xa monitoring in CKD stage 5 and dialysis and two nephrology doctors not undertaking monitoring.

### VTE prophylaxis

There were 79 respondents who answered the question relating to VTE thromboprophylaxis. Of respondents stating the dose, 81% in CKD stage 4 up to 88% in dialysis, used reduced doses of LMWH for VTE prophylaxis. Reduced doses included 20 mg enoxaparin, 2500units dalteparin and 2500 or 3500units of tinzaparin, supplementary Fig. 2. A small proportion, 5%, of respondents switched to using subcutaneous UFH for patients on dialysis.

### The use of prophylactic anticoagulation in nephrotic syndrome

There were four haematologists, 28 nephrologists and 24 pharmacists who responded that they use prophylactic anticoagulation for patients with NS. The majority of respondents, 93%, would use serum albumin as a factor in determining whether to use prophylactic anticoagulation, in addition 89% would take into consideration bleeding risks. A further 61% of respondents would use the degree of proteinuria (level not specified) and 50% would consider the primary cause of NS in their decision making.

The choice of agents used for prophylaxis in NS are shown in supplementary Fig. 3. For those with an albumin < 20 g/dL all respondents would administer anticoagulant therapy. This was more likely to be therapeutic anticoagulation with LMWH, warfarin or DOACs than those with higher levels of albumin. Once albumin increased above 25 g/dL then anticoagulation was less likely, with 54% respondents not using any anticoagulation. From the four centres where multi-professional groups responded, nephrology doctors suggested use of DOACs off-label in nephrotic syndrome which was not suggested as an option by their haematology and renal pharmacist counterparts. In two centres there was variation between nephrology doctors in the options considered for anticoagulation in people with nephrotic syndrome.

### Anticoagulant use in patients with AF and CKD

In terms of risk scores CHA_2_DS_2_-VASc (Congestive heart failure = 1, Hypertension = 1, Age > 65 = 1, Age > 75 = 2, Diabetes = 1, Stroke/TIA/Thromboembolism history = 2, Vascular disease = 1, Female sex = 1) [25] was used by 90% respondents (*N* = 64) to decide upon anticoagulant initiation for AF in patients with CKD. The bleeding scores HAS-BLED (Hypertension = 1, Age > 65 = 2, Stroke history = 1, renal disease = 1, Liver disease = 1, labile INR = 1, ethanol = 1, drugs = 1) [26] and ORBIT [27] being used by 78% and 8% respondents respectively. 10% of respondents did not use any risk scores when making anticoagulant decisions, supplementary Fig. 4.

Respondents were more likely to anticoagulate patients with CKD stage 4 than those on dialysis, Supplementary Fig. 4. Those on dialysis were more likely to be discussed as part of the MDT or as part of individualised decision making when initiating therapy. HASBLED > 3 influenced the decision-making process around anticoagulation for all stages of CKD, Supplementary Fig. 5.

**Fig. 4:**
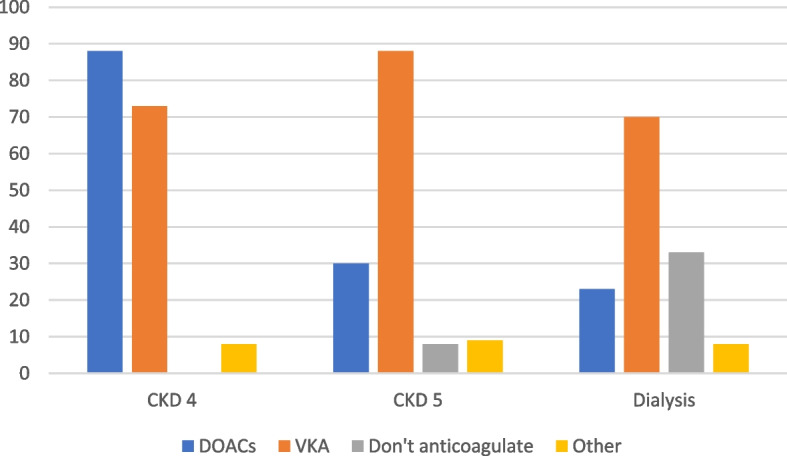
Anticoagulant options selected for AF dependant on CKD stage. DOACs = Direct-acting oral anticoagulant, VKA = vitamin K antagonist

For patients with AF and CKD stage 4, 88% of respondents would use DOACs for anticoagulation which reduced to 30% of respondents in those with CKD stage 5 and 23% in dialysis patients, Fig. 4. In two centres with multiple nephrology doctor respondents there was variation between clinicians with some considering the use of DOACs in CKD stage 5 and dialysis but not others. From the four centres where multiple professional groups responded, nephrology doctors used DOACs for AF in CKD stage 5 and dialysis, in contrast to haematology and renal pharmacists who did not consider them options. In CKD stage 5 and dialysis, VKAs were the main anticoagulant therapy used. A third of respondents (33%) suggested they wouldn’t anticoagulate people on dialysis with AF. The respondents who did not advocate anticoagulation were from nine regions of the United Kingdom and fourteen different hospital trusts. There was in-centre variation with nephrology doctors in three centres, having respondents that wouldn’t use anticoagulation for people with AF on dialysis and those that would anticoagulate.

Apixaban was the most frequent DOAC prescribed being used by 74% of respondents in CKD stage 4. For those who used DOACs in CKD stage 5 and dialysis, 87% and 81% used apixaban respectively with the remainder using edoxaban.

There were 48 respondents to the question whether DOAC level monitoring was undertaken. Only one respondent (2%) would routinely check DOAC levels in CKD stage 4, 19% in CKD stage 5 and 15% in dialysis patients.

## Discussion

This was the first study examining national prescribing practice of anticoagulants in patients with CKD across specialities within the UK.

The main findings from this survey include (1) a lack of multidisciplinary groups supporting anticoagulant use in CKD (2) variations in the use of renal function estimating equations for drug dosing (3) choice of anticoagulant for VTE varies depending on the speciality initiating treatment (4) heterogeneity in anticoagulant used, dosing and monitoring across the indications and stages of CKD (5) main considerations around use of anticoagulants in NS includes serum albumin and bleeding risk (6) DOACs are being used off-licence for the thromboembolic reduction of AF in patients with CKD stage 5 and on dialysis.

Only 3% of respondents reported having an MDT for discussing anticoagulant use in patients with CKD despite decision-making being challenging in this population. This finding is similar to a European survey of nephrologists and cardiologists managing patients with CKD and AF [28]. Previously the impact of MDTs on treatment of patients with VTE found that the duration and assessment for therapeutic/prophylactic was significantly changed [29].

### Renal function estimate for dosing of anticoagulants

There are variations in the use of renal estimating equations for anticoagulant dosing between pharmacists, haematology and nephrology doctors. The manufacturers of fondaparinux, LMWHs and DOACs all recommend the use of C-G CrCl for dosing based on how renal function was estimated in the clinical trials. In the mainstay, this is undertaken by pharmacists and haematologists but less so nephrologists. Similar findings were also seen in the recent ERA-EHRA survey where the majority of nephrologists would use alternate renal function estimates whilst cardiologists used C-G CrCl [28]. The Medicines Healthcare Regulatory Authority (MHRA) has issued guidance recommending the use of C-G CrCl for DOAC dosing due to misclassification of dosing if other equations are used [30]. This was shown in a study by Kruger et al., which compared C-G CrCl to CKD-EPI and MDRD in patients taking DOACs [31]. In this study, 48% of patients with a CrCl < 30 ml/min and 46% of patients with a CrCl of 30–49 ml/min would have received treatment inconsistent with current guidelines if MDRD or CKD-EPI had been used [31]. Nephrologists are familiar with using CKD-EPI due to it being validated for estimating renal function in wide range of patient populations [32], unlike C-G formula. This may be a factor in nephrology doctors dosing decisions, but further research is warranted to determine whether a body surface area adjusted eGFR is suitable for use in the dosing of DOACs.

### Treatment of VTE

In acute VTE, no respondents used intravenous (IV) UFH in patients with CKD stage 4 whilst eight centres used IV UFH in those on dialysis. The use of UFH is time-consuming for nursing staff as it requires monitoring and complex dose regimes which is probably why it has gone out of favour. However due to a lack of literature around dosing of LMWH in advanced kidney disease, UFH may be selected to avoid under and over-dosing of a LMWH. The European Society of Cardiology and European Respiratory Society guidelines for PE suggest using UFH in those with CKD stage 5 or on dialysis [33]. Interestingly, units using LMWH for CKD stage 4 through to dialysis, reduce the dose to two thirds of the original licenced dose. This regime was published in a practical paper on anticoagulation in CKD in 2014 based on author expertise [34]. The National Institute for Clinical Excellence (NICE) suggest when using LMWH treatment for VTE in CrCl < 15ml/min, that the monitoring and dosing should follow manufacturers information and locally agreed protocols [35]. Over 30% respondents do not undertake anti-Xa monitoring with therapeutic LMWH in CKD stages 5 and dialysis. When using therapeutic doses of LMWH in patients with severe renal impairment CrCl < 30ml/min, anti-Xa level monitoring should be considered [36–38] with dose adjustments as appropriate [34, 35] to avoid over and under dosing.

For long-term management of VTE, all professional groups were more likely to use DOACs than LMWH or VKAs for patients with CKD stage 4. This finding would align with current NICE guidelines for VTE diseases [35]. DOACs have reduced monitoring requirements and a simple dosing regime, which make them an appealing option to patients and clinicians. For patients with CKD stage 5 and on dialysis, most respondents would use VKAs which is likely due to a lack of licensing and limited published evidence for DOACs in this scenario. Apixaban was the DOAC selected by all respondents when used in CKD stage 5 and dialysis and this may be based on the findings of a number of observational studies and a recent systematic review from our group which showed apixaban had a lower risk of bleeding compared to warfarin [9, 39–44]. However, more evidence is needed to support the use of DOACs in the treatment of VTE in patients with CKD stage 5 and dialysis.

In renal transplant recipients, most renal pharmacists and nephrology doctors would use DOACs. Haematologists were more conservative with only 58% of them opting to use DOACs. Reluctance to use DOACs may relate to the current EHRA guidelines which suggest consider avoiding DOACs with tacrolimus due to a potential interaction [45], with tacrolimus being used in most transplant recipients. However, a recent study suggests that tacrolimus has a limited impact on apixaban and rivaroxaban levels and they may be considered as an anticoagulant option [46].

### VTE prophylaxis

For patients with CKD stage 4, the majority of respondents suggested they would use a reduced dose of LMWH for VTE prophylaxis. This is despite only the manufacturers of enoxaparin suggesting a dose reduction is required with a CrCl < 30ml/min [38]. As patients progressed to CKD stage 5 and dialysis, there was increased use of UFH which may relate to uncertainty in dosing of LMWH. To date there are only very small studies with limited patients that suggest dalteparin and tinzaparin do not accumulate at prophylactic doses based on anti-Xa level monitoring [47]. These studies are short in duration so may not reflect what would happen with more prolonged administration.

### Anticoagulation in nephrotic syndrome

Over 80% of respondents use serum albumin and bleeding risk to consider the use of anticoagulation in NS. This practice corresponds with the recommendations in the KDIGO glomerular disease guide [48]. Kidney Disease Improving Global Outcomes (KDIGO) also suggest proteinuria > 10 g/day should be considered a risk factor based on a study by Kumar et al [49], with 60% of respondents considering the degree of proteinuria in their anticoagulation decision-making. The majority of recommendations in KDIGO relate to membranous nephropathy [48] although the primary reason for nephrotic syndrome was only a factor in decision making by 50% of respondents. The risks factors for VTE occurrence in NS are not yet fully elucidated which may explain the variability seen in what respondents use for decision making.

The choice of anticoagulant was mainly warfarin or therapeutic LMWH for patients with an albumin < 20 g/dL which are suggested options within KDIGO [48]. However, 25% of respondents would use a DOAC in patients with albumin < 20 g/dL which has currently been poorly studied and is limited to a single report of two cases. There is an ongoing study to determine apixaban pharmacokinetics in patients with NS taking multiple doses compared to healthy volunteers (NCT04278729).

### Anticoagulation in AF

When making decisions around anticoagulation for AF in patients with CKD most respondents would use the CHA2DS2-VASc and HAS-BLED risk scores. One in ten respondents did not use any risk scores and this may relate to their lack of validation in patients with advanced CKD [50, 51]. Respondents were less likely to anticoagulate patients on dialysis which may relate to the uncertainty in whether anticoagulation reduces the risk of stroke in this group [9, 52]. The use of anticoagulants versus no anticoagulation in dialysis patients with AF is currently being investigated in a number of RCTs [53–55]. Over 20% of respondents use DOACs for AF in patients with CKD 5 and on dialysis, which is mainly apixaban. DOACs are not licensed in this population in Europe, but there is increasing observational data that apixaban has a lower risk of bleeding compared to warfarin [56–59] and this may be influencing prescribing decisions. DOAC monitoring is not widely undertaken and may relate to the limited availability of monitoring.

### Limitations

This survey was based in the UK which limits the generalisability of findings to other countries. Fewer responses from haematology professionals and centres in Ireland and Wales may limit the generalisability of the findings for these nations. However, a wide geographical location of responses was obtained from the rest of the UK which is a strength of the survey. Response rates from nephrology doctors and haematology cannot be calculated, so this survey can only provide a limited representation of current practice. Voluntary participation may have led to some selection bias. There is potential for response bias as respondents may state they do something that they do not actually do in practice. However, giving respondents the option to remain anonymous should have supported professionals to feel comfortable being open about their practice.

## Conclusion

Some consistency is required around the use of renal function estimating equations for the dosing of anticoagulants. DOACs are being used in a number of off-label scenarios including treatment of VTE and AF in patients with end-stage renal disease as well as active nephrotic syndrome. More evidence is needed to determine whether DOACs are the appropriate treatments in these situations. A best-practice guideline would be useful to support anticoagulant practice across the UK. The use of the Delphi technique with experts, has been used in such situations of limited evidence, to support development of best-practice guidelines.

## Supplementary Information

**Additional file 1.**

**Additional file 2.**

**Additional file 3.**

**Additional file 4.**

## Data Availability

The majority of data is provided in the supplementary appendices but any further data underlying this article will be shared on reasonable request to the corresponding author.
