# Supplementary material for: UK prescribing practice of anticoagulants in patients with chronic kidney disease: a nephrology and haematology-based survey
Source: BMC Nephrol. 2023 Jan 12;24:9. doi: 10.1186/s12882-022-03041-w (PMC9837988; doi:10.1186/s12882-022-03041-w)
Supplement: Supplementary file 1 — Additional file 1. [file 12882_2022_3041_MOESM1_ESM.docx]

Supplementary Appendix 1. Survey questions

Part 1 General questions

1. What is your job role?

Doctor in haematology

Doctor in nephrology

Nurse in nephrology

Advanced nurse practitioner in nephrology

Pharmacist in nephrology

Other please specify

2. As part of your job role would you see any of the below patients (tick all that apply)

CKD stage 4 (eGFR15-29ml/min) patients with acute VTE

CKD Stage 5 not on dialysis (eGFR<15ml/min) patients with acute VTE

Dialysis patients (PD or HD) patients with acute VTE

CKD stage 4 (eGFR15-29ml/min) patients with AF

CKD Stage 5 not on dialysis (eGFR<15ml/min) patients with AF

Dialysis patients (PD or HD) patients with AF

CKD stage 4 (eGFR15-29ml/min) patients requiring medical VTE prophylaxis

CKD Stage 5 not on dialysis (eGFR<15ml/min) patients requiring medical VTE prophylaxis

Dialysis patients (PD or HD) patients requiring medical VTE prophylaxis

Patients with kidney transplant

Patients with nephrotic syndrome

3. Does your centre have a structured multidisciplinary team to manage patients with CKD on anticoagulants?

Yes please list specialities included

No

4. How would you assess renal function in the context of prescribing the following anticoagulants in CKD (select one per anticoagulant class)

Cockcroft-gault MDRD CKD-EPI OTHER

DOACS

LMWH

Fondaparinux

Unfractionated heparin

Argatroban

VKA

Part 2. VTE Prophylaxis

5. What do you routinely use for pharmacological VTE prophylaxis in CKD stage 4 patients? (please specify dose and frequency)

Enoxaparin

Dalteparin

Tinzaparin

Unfractionated heparin

Other

6. What do you routinely use for pharmacological VTE prophylaxis in CKD stage 5 patients? (please specify dose and frequency)

Enoxaparin

Dalteparin

Tinzaparin

Unfractionated heparin

Other

7. What do you routinely use for pharmacological VTE prophylaxis in dialysis patients? (please specify dose and frequency)

Enoxaparin

Dalteparin

Tinzaparin

Unfractionated heparin

Other

8. If a patient with CKD was unable to have heparins what option would you consider for VTE prophylaxis?

CKD stage 4 CKD stage 5 Dialysis

Fondaparinux

DOAC

Mechanical prophylaxis

Other please specify

Part 3. Management of Acute VTE in CKD

9. For patients with CKD which options would you use for acute phase treatment of VTE? (select all that apply)

CKD stage 4 CKD stage 5 Dialysis Kidney transplant

LMWH

DOAC

IV UFH

Part b If you use LMWH for VTE treatment which LMWH and what dose do you use?

CKD stage 4

CKD stage 5

Dialysis

Part c If you use LMWH for VTE treatment would you monitor:

CKD stage 4 CKD stage 5 Dialysis

Peak anti-xa

Trough anti-xa

Both

Wouldn’t monitor

10. For patients with CKD which options would you use for chronic treatment of VTE? (select all that apply)

CKD stage 4 CKD stage 5 Dialysis Kidney transplant

LMWH

DOAC

VKA

Part b if you use DOACs for treatment of VTE which DOAC and what dose would you use?

CKD stage 4

CKD stage 5

Dialysis

Part 4. Management of AF in CKD

11. Do you routinely use risk scores to aid your decision making when anticoagulating CKD patients with AF

HASBLED

CHADSVASc

Both

Other (please state)

Don’t use risk scores

12. Would you anticoagulate pt with CHADSVASC>2 with HASBLED<3

CKD stage 4 CKD stage 5 Dialysis

Yes

No

Not sure

Discuss at MDT

Individual decision made with patient

13. Would you anticoagulate a patient with CHADSVASC>2 with HASBLED>3

CKD stage 4 CKD stage 5 Dialysis

Yes

No

Not sure

Discuss at MDT

Individual decision made with patient

14. Would you anticoagulate a patient with CHADSVASC>5 with HASBLED>3

CKD stage 4 CKD stage 5 Dialysis

Yes

No

Not sure

Discuss at MDT

Individual decision made with patient

15. What anticoagulants would you use for CKD patients with AF?

CKD stage 4 CKD stage 5 Dialysis Kidney transplant

DOACs

VKA

Other

Not applicable

16. If you use DOACs in patients with CKD and AF what DOAC and dose do you use?

CKD 4

CKD 5

Dialysis

Part b. Would you check or monitor DOAC levels?

Yes- All patients Yes-In some patients No

CKD 4

CKD 5

Dialysis

Kidney transplant

Part 5- Nephrotic syndrome

17. What factors do you consider when making decisions about anticoagulation in nephrotic syndrome? (Select all that apply)

Degree of proteinuria

Serum albumin

Primary renal condition

Bleeding risk

Others, please state

18. What options would you use for anticoagulation?

Serum albumin <20g/dL 20-25g/dL 26-30g/dL

DOAC

LMWH therapeutic

LMWH prophylaxis

Aspirin

Warfarin

None

Other, please state

Please state where you work :

(This is for the purpose of identifying multiple responses from the same centre only and will not be used for any other purpose. This information will be removed and discarded prior to data analysis)

(Non- compulsory question) If you are happy to be contacted at a later date for further involvement in this research please provide your contact details here:
