# Supplementary material for: UK prescribing practice of anticoagulants in patients with chronic kidney disease: a nephrology and haematology-based survey
Source: BMC Nephrol. 2023 Jan 12;24:9. doi: 10.1186/s12882-022-03041-w (PMC9837988; doi:10.1186/s12882-022-03041-w)
Supplement: Supplementary file 2 — Additional file 2. [file 12882_2022_3041_MOESM2_ESM.docx]

Supplementary Figure 2. Agents and dose used for Venous thromboembolism prophylaxis

*all doses are once daily unless otherwise specified.

VTE= venous thromboembolism, UFH= Unfractionated Heparin, bd= twice daily, tds= three times daily, CKD= chronic kidney disease

Supplementary Figure 3. Choice of prophylactic anticoagulant in nephrotic syndrome stratified by albumin

DOACs= Direct acting oral anticoagulant, LMWH= Low Molecular Weight Heparin

Supplementary Figure 4. Risk scores used in anticoagulant decisions for Atrial Fibrillation in Chronic Kidney Disease

HAS-BLED (Hypertension = 1, Age>65=2, Stroke history=1, renal disease= 1, Liver disease= 1, labile INR=1, ethanol=1, drugs=1), CHA_2_DS_2_-VASc (Congestive heart failure=1, Hypertension=1, Age>65 = 1, Age>75=2, Diabetes= 1, Stroke/TIA/Thromboembolism history= 2, Vascular disease= 1, Female sex= 1), CHADS_2_ = (Congestive heart failure=1, Hypertension=1, Age>65 = 1, Diabetes= 1, Stroke/TIA/Thromboembolism history= 2), ORBIT = (older>75years =1. Reduced haemoglobin (<13 mg/dL in men and <12 mg/dL in women), haematocrit (<40% in men and <36% in women) or history of anaemia =2, bleeding history=2, insufficient kidney function (eGFR < 60 mg/dL/1.73 m2) =1, treatment with an antiplatelet agent =1)

Supplementary Figure 5. Decisions around anticoagulation based on CKD stage and risk scores

From left to right CKD stage 4, CKD stage 5 and dialysis

Chronic Kidney Disease =CKD, Multidisciplinary Team = MDT, CHA2DS2-VASc (Congestive heart failure=1, Hypertension=1, Age>65 = 1, Age>75=2, Diabetes= 1, Stroke/TIA/Thromboembolism history= 2, Vascular disease= 1, Female sex= 1), HAS-BLED (Hypertension = 1, Age>65=2, Stroke history=1, renal disease= 1, Liver disease= 1, labile INR=1, ethanol=1, drugs=1),
