# Supplementary material for: UK prescribing practice of anticoagulants in patients with chronic kidney disease: a nephrology and haematology-based survey
Source: BMC Nephrol. 2023 Jan 12;24:9. doi: 10.1186/s12882-022-03041-w (PMC9837988; doi:10.1186/s12882-022-03041-w)
Supplement: Supplementary file 3 — Additional file 3. [file 12882_2022_3041_MOESM3_ESM.docx]

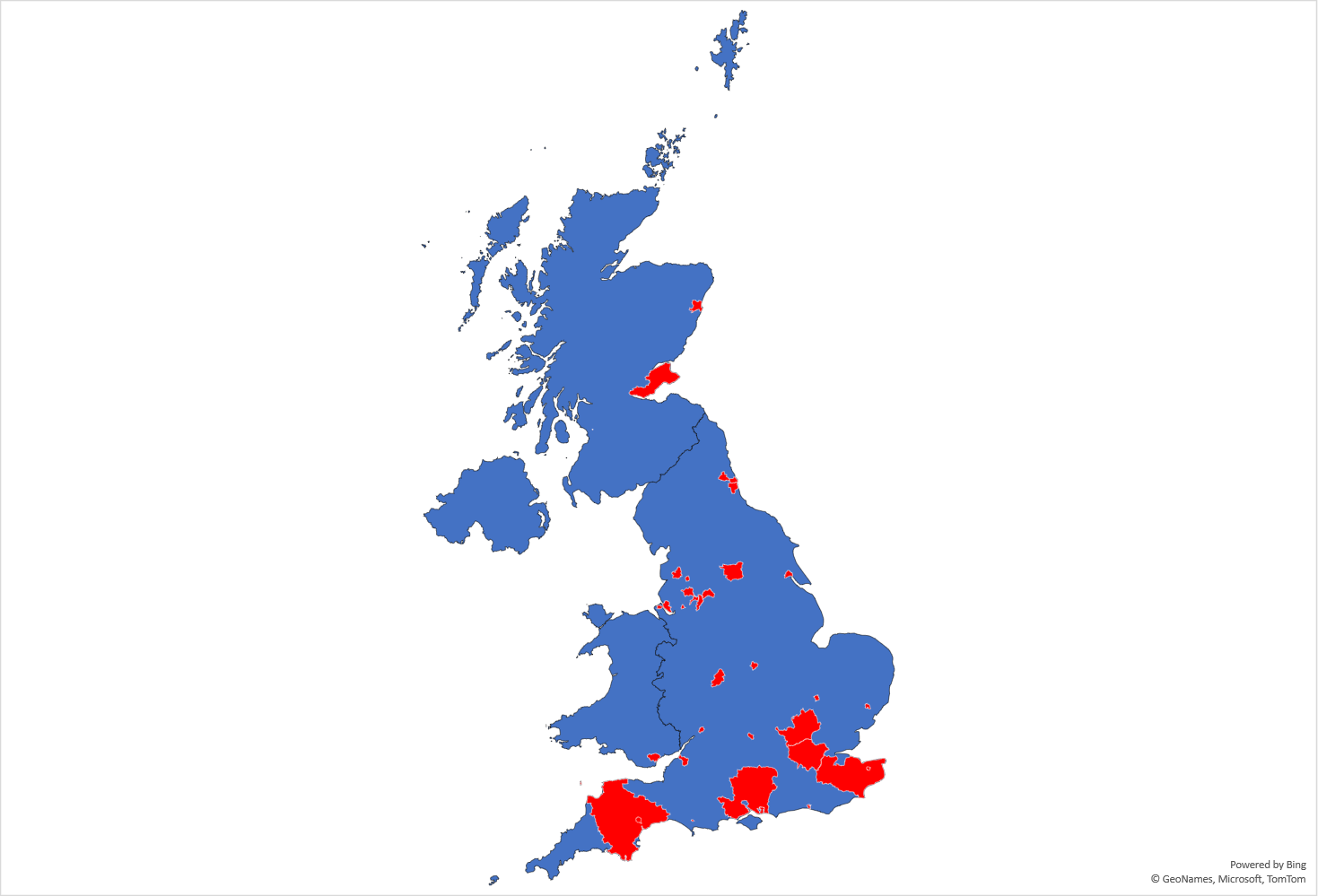
Supplementary Figure 1. Location of survey responses

Red indicates areas where responses have been received

Supplementary table 1. Location of survey responses by region

| Region | No. respondents | No. Centres responded |
| --- | --- | --- |
| Scotland | 3 | 3 |
| Wales | 1 | 1 |
| Northern Ireland | 1 | 1 |
| East of England | 5 | 3 |
| North West England | 28 | 9 |
| North East England | 2 | 2 |
| West Midlands | 1 | 1 |
| East Midlands | 1 | 1 |
| Yorkshire and the Humber | 3 | 2 |
| London | 4 | 3 |
| South East England | 10 | 5 |
| South West England | 4 | 4 |
| Anonymous responses | 46 |  |
