# Supplementary material for: UK prescribing practice of anticoagulants in patients with chronic kidney disease: a nephrology and haematology-based survey
Source: BMC Nephrol. 2023 Jan 12;24:9. doi: 10.1186/s12882-022-03041-w (PMC9837988; doi:10.1186/s12882-022-03041-w)
Supplement: Supplementary file 4 — Additional file 4. [file 12882_2022_3041_MOESM4_ESM.docx]

Supplementary table 2

Direct oral-acting anticoagulants used in the treatment of Venous thromboembolism

|  | CKD stage 4 | CKD stage 5 | Dialysis |
| --- | --- | --- | --- |
| Nephrology doctors | Apixaban 2.5mg bd n =10  Apixaban 2.5-5mg bd n= 2  Apixaban 2.5-5mg bd after loading n=1  Apixaban 5mg bd n=5  Edoxaban 30mg od n=2  Apixaban as per BNF/SPC n=2  Apixaban or rivaroxaban n=1  Apixaban n=3  Apixaban as per AF dosing n=1 | Apixaban 2.5mg bd n=10  Apixaban 2.5mg bd after loading n=1  Apixaban 5mg bd n=1  Apixaban as per AF criteria n=1  Apixaban n=3 | Apixaban 2.5mg bd n= 6  Apixaban 5mg bd n= 1  Apixaban 2.5mg bd after loading n=1  Apixaban n=3  Apixaban as per BNF n=1  Apixaban as per AF criteria n=1 |
| Pharmacists | Rivaroxaban n=2  Apixaban n=11  Edoxaban n=2  Apixaban 2.5mg bd n=5  Edoxaban 30mg od n=6  Apixaban 5mg bd n=8  Rivaroxaban 15mg bd n=5 | Apixaban 5mg bd n=1  Apixaban 2.5mg bd n=1  Apixaban n=3 | Apixaban n=2  Apixaban 2.5mg bd n=1 |
| Haematologists | Apixaban 5mg bd n=1  Edoxaban 30mg od n=2  Apixaban n=3  Rivaroxaban n=1 | Not stated n=11 | Not stated n=1 |

od= once daily, bd = twice daily, BNF = British National Formulary, SPC = Summary of Product Characteristics, AF = Atrial Fibrillation
